# A lightweight attention-enhanced framework for crop pest and disease detection in desert greenhouses

**DOI:** 10.3389/fpls.2026.1830048

**Published:** 2026-07-15

**Authors:** Yunsen Liang, Kewen Ouyang, Zijing Luo, Naimin Kong, Chi Qin, Yaoxue Xu, Chan Zhang, Zhiyuan Zou, Yining Zhang, Mingman Xu, Siqin Zhong, Chengyao Jiang, Mengyao Li, Yangxia Zheng, Dawei Shi, Sen Wang, Chengbo Zhou, Qichang Yang, Wei Lu

**Affiliations:** 1 College of Information Engineering, Sichuan Agricultural University, Ya’an, China; 2 College of Horticulture, Sichuan Agricultural University, Chengdu, China; 3 College of Electrical and Mechanical Engineering, Sichuan Agricultural University, Ya’an, China; 4 Institute of Urban Agriculture, Chinese Academy of Agricultural Sciences, Chengdu, China

**Keywords:** agricultural pest and disease detection, attention mechanism, deep learning, desert greenhouse, lightweight detection, YOLO-based framework

## Abstract

Desert greenhouse environments present challenging visual conditions for crop pest and disease detection because of strong illumination, reflective backgrounds, and frequent leaf occlusion. To address these issues, we propose YOLOv11s-DesertAttn, a lightweight detection framework that integrates ADown, SimAM, and LSKAttention to improve feature preservation, background suppression, and high-level semantic representation. On the test set, the proposed model improves mAP@0.5–0.95 from 0.826 to 0.860 while reducing parameters from 9.43 M to 8.14 M and increasing inference speed from 96 FPS to 116 FPS. Additional evaluation on an independent real-world dataset collected from commercial greenhouses in Hotan, Xinjiang further demonstrates the model’s robustness under varying illumination and occlusion conditions, indicating its potential for real-time deployment in intelligent greenhouse monitoring systems.

## 1 Introduction

Facing the dual challenges of global climate change and the scarcity of cultivated land resources, utilizing non-arable lands such as Gobi deserts and sandy areas to develop facility agriculture has become a major strategic necessity for ensuring food security and expanding agricultural space (Sharma et al., 2023). However, the unique geographical location of desert greenhouses creates an exceptionally distinctive microclimate—characterized by extreme diurnal temperature variations, intense solar radiation, and low humidity (Nicola et al., 2020; Campana et al., 2025). This harsh environment not only predisposes crops to physiological stress but also fundamentally alters the occurrence and evolution patterns of pests and diseases. The highly enclosed nature and high-density planting within the greenhouse mean that once a pest or disease outbreak occurs, it can lead to devastatingly rapid spread (Zheng and Xu, 2023). Therefore, achieving early and precise automated monitoring of pests and diseases is a prerequisite for safe production in desert greenhouses. In recent years, with the rapid advancement of computer technology, deep learning-based automatic crop disease detection techniques have demonstrated significant advantages (Wani et al., 2022). However, the highly reflective background and strong heterogeneous lighting inside desert greenhouses severely interfere with the visual feature extraction of conventional models, leading to a sharp decline in detection performance (Akbar et al., 2024). How to achieve efficient pest and disease detection under such extreme optical conditions in desert greenhouses has become a critical challenge urgently needing resolution.

To address the interference of complex backgrounds and lighting conditions on pest and disease detection, existing research has conducted extensive explorations. For instance, Zhang et al. (2025) proposed an improved recognition model based on MobileViT, achieving a good balance between lightweight design and detection accuracy (Zhang et al., 2025); Yan et al. (2025), targeting the small-target detection problem, introduced a specific down-sampling module to construct the YOLO-OS model, effectively enhancing target detection accuracy (Yan and Feng, 2025). However, existing detection models and conventional attention mechanisms are mostly developed based on traditional open-field or standard greenhouse environments, making it difficult for them to effectively suppress the high-frequency noise induced by the unique strong light interference and high background similarity in desert greenhouses (Wu et al., 2023). Introducing semantic enhancement and anti-aliasing techniques to strengthen feature extraction capabilities offers new ideas for overcoming the interference of these harsh environments (Zou et al., 2023). Furthermore, images captured in the high-density planting mode of desert greenhouses often contain numerous small-scale lesions in the early stages of infection, accompanied by severe mutual occlusion of leaves, further compounding the detection difficulty (Dang et al., 2024; Reddy, 2024). To tackle the challenge of small-target detection, some recent studies have attempted to alleviate missed detections in agricultural images containing multiple small targets through multi-scale feature fusion and network structure adjustments (Wang et al., 2022). Most of these methods adopt a traditional strategy of stacking complex computational modules, which, while pursuing detection accuracy, sacrifices model size and computational cost (FLOPs), making them difficult to deploy on resource-constrained Internet of Things (IoT) mobile terminals or edge devices (Shuvo et al., 2022). Recent studies have further highlighted that lightweight detector design should be evaluated not only by accuracy but also by deployment feasibility on embedded platforms. For example, Dong et al. (2025) developed a lightweight rail defect detection network and demonstrated practical edge-side deployment using NVIDIA Jetson Nano and TensorRT acceleration, showing that compact architecture design, efficient feature fusion, and deployment-oriented evaluation are critical for real-time industrial inspection systems (Dong et al., 2025a). Although their task focuses on rail defect detection rather than agricultural disease detection, their study provides important evidence that lightweight visual detectors can achieve effective real-time performance in resource-constrained edge environments, which is highly relevant to intelligent greenhouse monitoring applications. Therefore, constructing models that are both lightweight and capable of accurately detecting occluded small-scale pest and disease targets in extreme greenhouse scenarios remains a focal point of current global research (Albahli, 2025).

Recently, several studies published in 2025–2026 have further improved lightweight object detection by optimizing model structures and reducing computational redundancy (Luo et al., 2025). In addition, research on complex visual environments has explored more robust feature extraction strategies to enhance performance under occlusion and illumination variations (Dong et al., 2025b).

However, these methods are primarily designed for general scenarios and lack targeted optimization for desert greenhouse conditions, where strong light reflection, plastic film interference, and dynamic illumination significantly increase detection difficulty. This gap motivates the development of a scenario-specific lightweight detection framework tailored to such environments.

In view of this, this study aims to develop an efficient pest and disease detection model suitable for extreme desert greenhouse scenarios, with a focus on improving the detection performance of small-scale pest and disease targets under strong light interference. Leveraging a self-constructed, highly diverse desert greenhouse dataset, this paper designs a lightweight YOLOv11s-DesertAttn detection framework that balances high detection accuracy, small parameter count, and low computational cost. The contributions of this study are as follows: (1) a multi-source pest and disease dataset containing 38,371 annotated images across 38 fine-grained categories and an additional independent real-world test set of 4,328 images collected from commercial desert greenhouses in Hotan, Xinjiang were constructed; (2) a lightweight anti-interference detection framework integrating ADown, SimAM, and LSKAttention was developed; (3) the hierarchical design improved the representation of small and partially occluded targets under complex illumination; and (4) the resulting model achieved a favorable balance between detection accuracy, computational cost, and deployment efficiency.

Unlike prior studies that mainly focus on standard greenhouse or open-field conditions, this study consistently defines the task as crop pest and disease detection in desert greenhouse environments. Throughout the manuscript, the term detection is used to describe the target task. Expressions such as small-scale pest and disease targets, lesion regions, and occluded targets are only used to describe specific visual manifestations or detection challenges within this unified task, rather than different task definitions. This terminology is adopted consistently in the Introduction, Methods, Results, and Conclusions to avoid ambiguity.

## 2 Materials and methods

### 2.1 Overview of the model improvements

YOLOv11 is an object detection model further optimized based on YOLOv8 (Xu et al., 2025). Its overall architecture consists of an input module, a backbone network (Backbone), a neck (Neck), and a detection head (Head). Compared with other models in the YOLO series, YOLOv11 demonstrates improvements in both detection accuracy and inference efficiency, primarily attributable to systematic optimizations in the feature extraction structure, feature fusion mechanism, and detection head design.

**Figure 1:**
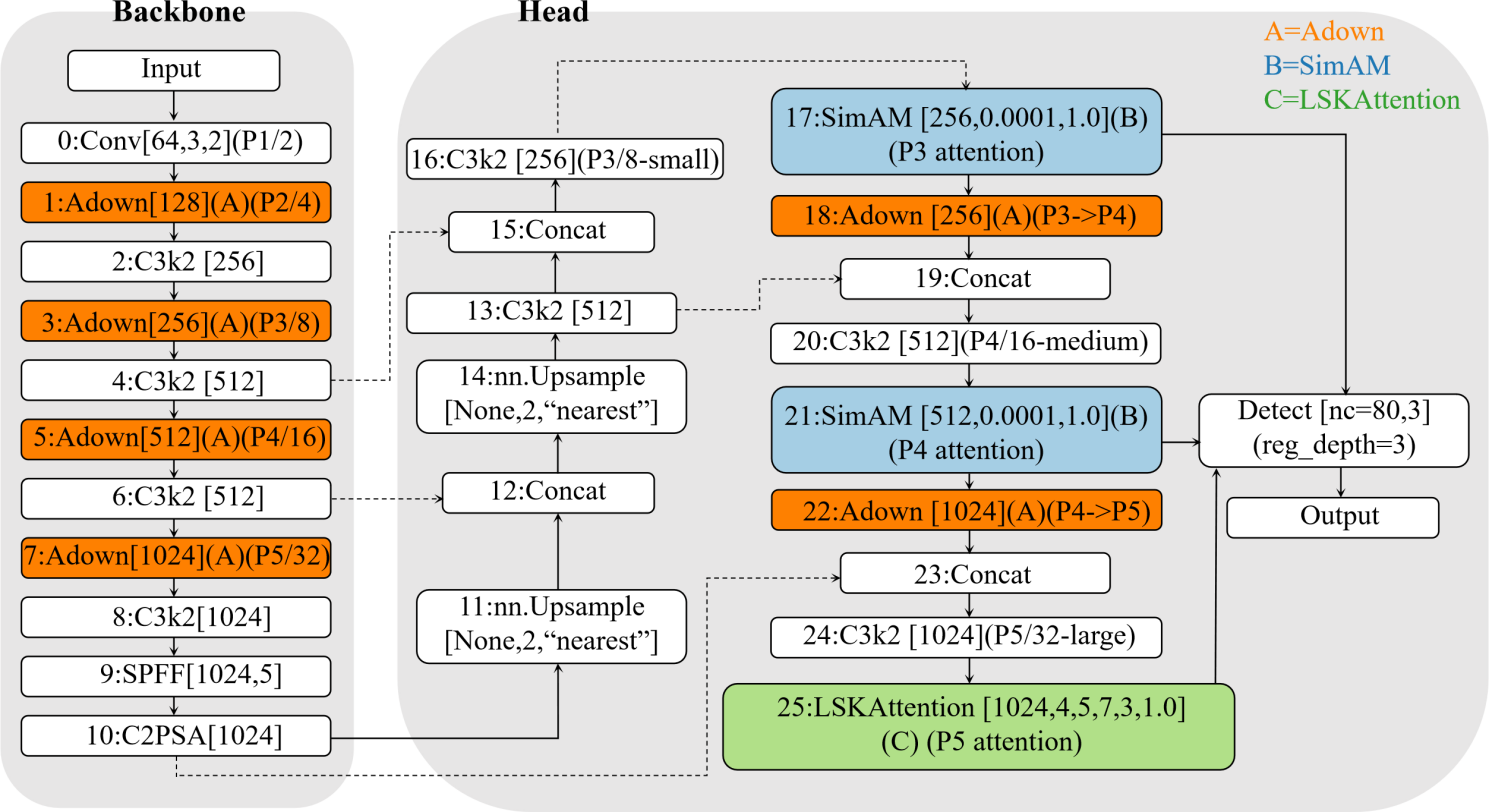
Architecture of the YOLOv11s-DesertAttn module.

To address practical challenges in desert greenhouse environments—such as small-scale pest and disease targets, complex backgrounds, and strong yet uneven illumination—while ensuring model real-time performance and computational efficiency, it is necessary to enhance the fine-grained feature preservation capability for small targets, the discriminative ability under complex background conditions, and the stability of multi-scale feature representation (Wang et al., 2025). Therefore, this study selects YOLOv11s as the base model and conducts structural optimization centered on its key components. The overall architecture of the proposed YOLOv11s-DesertAttn model is illustrated in **Figure 1**. A lightweight detection model tailored to desert greenhouse application scenarios, termed YOLOv11s-DesertAttn, is proposed.

Specifically, the improvement strategy is carried out from three aspects: down-sampling mechanism optimization, feature recalibration enhancement, and high-level semantic modeling, which are respectively applied to the Backbone and Neck stages. First, during the feature down-sampling process, to mitigate the aliasing effect and detail information loss potentially caused by traditional stride = 2 convolution, an anti-aliasing down-sampling module (ADown) is introduced to replace the original down-sampling structure. This module adopts a pooling-dominant spatial down-sampling approach, which suppresses high-frequency information folding while reducing feature resolution, thereby improving the consistency and stability of multi-scale feature construction (Xiang et al., 2022).

Second, at the intermediate feature stage, a parameter-free attention mechanism, SimAM, is introduced to perform adaptive recalibration on high-resolution feature layers within the Neck, where semantic information is progressively enhanced. This mechanism evaluates the importance of neurons based on an energy function and strengthens the response of target regions while suppressing background interference, without significantly increasing the number of parameters or computational complexity (Yu et al., 2024).

At the high-level semantic feature stage, a Large Selective Kernel Attention module (LSKAttention) is incorporated. By constructing multi-scale receptive fields and performing selective feature fusion, this module enhances the model’s capability to capture large-scale disease regions and continuous structural targets, thereby improving semantic representation performance under complex greenhouse conditions (Du and Liang, 2024).

The proposed improvement strategy is hierarchically deployed at different levels of the network, systematically optimizing the YOLOv11 framework in terms of down-sampling stability, feature recalibration capability, and receptive field expansion. While maintaining a compact model structure, the proposed approach achieves a coordinated balance among detection accuracy, computational complexity, and inference speed, thereby providing technical support for real-time monitoring and intelligent management of crop pests and diseases in desert greenhouse environments.

The placement of the three modules was determined according to the functional characteristics of different feature levels. ADown was introduced at key down-sampling transitions to reduce aliasing and preserve fine lesion details during spatial resolution reduction. SimAM was inserted at the P3/P4 levels because these feature maps retain relatively high spatial resolution and are more sensitive to small pest targets and low-contrast lesion patterns under background interference. LSKAttention was applied at the P5 level to enhance high-level semantic aggregation and improve the modeling of large-area disease regions and continuous structural damage. This hierarchical deployment was designed to match the visual challenges of desert greenhouses, including strong illumination, reflective film backgrounds, and partial leaf occlusion.

### 2.2 Anti-aliasing Downsampling

In YOLO-based crop pest and disease detection networks, down-sampling operations constitute a critical step in constructing multi-scale feature representations. However, traditional stride = 2 convolution reduces spatial resolution while potentially introducing aliasing effects and causing the loss of fine-grained details. In desert greenhouse scenarios—characterized by intense and uneven illumination, strong reflections from greenhouse films, and small-scale lesion and insect targets—such information degradation is more likely to adversely affect detection performance.

To address these issues, this study introduces an Anti-aliasing Downsampling (ADown) module (Wang et al., 2024) to replace the standard down-sampling structure within the network. The ADown module was first proposed in YOLOv9. Related studies have demonstrated that it can effectively improve the stability of feature representations while maintaining computational efficiency. The core concept of ADown is to explicitly suppress aliasing effects during spatial resolution compression, thereby providing more reliable input features for subsequent feature fusion and object prediction stages.

Unlike traditional stride = 2 convolution, ADown employs pooling operators to dominate the spatial down-sampling process. While reducing resolution, it preserves local structural consistency, enabling the network to maintain higher sensitivity to fine-grained pest and disease features during multi-scale feature construction.

Specifically, let the input feature map be defined as:


$$\text{X}\in {\mathbb{R}}^{\text{H}\times \text{W}\times \text{C}}$$

The ADown module consists of two parallel down-sampling branches. The first branch applies a max-pooling operation with stride 2 to extract locally salient responses:


$${\text{X}}_{\text{max}}={\text{MaxPool}}_{2\times 2}(\text{X})$$

The second branch applies an average-pooling operation with stride 2 to preserve low-frequency contextual information:


$${\text{X}}_{\text{avg}}={\text{AvgPool}}_{2\times 2}(\text{X})$$

The outputs of the two branches are then concatenated along the channel dimension:


$${\text{X}}_{\text{cat}}=\text{Concat}({\text{X}}_{\text{max}},{\text{X}}_{\text{avg}})$$

Subsequently, a 1×1 convolution is applied to achieve channel fusion and compression, producing the final output feature:


$$\text{Y}={\text{Conv}}_{1\times 1}({\text{X}}_{\text{cat}})$$

In the proposed model, the ADown module is systematically deployed at key down-sampling positions within both the Backbone and Neck to ensure that the multi-scale feature pyramid maintains consistent anti-aliasing characteristics during resolution transitions. By preserving more structural information relevant to crop pests and diseases during resolution changes, ADown enhances the stability and discriminative capability of feature representations under complex desert greenhouse conditions.

Compared with standard stride = 2 convolution, ADown primarily consists of parameter-free pooling operations and a lightweight 1×1 convolution. The increase in parameters and computational cost is minimal. Replacing conventional sampling structures with ADown in agricultural object detection tasks can effectively improve the detection performance of small-scale and densely distributed targets with negligible additional inference overhead.

**Figure 2:**
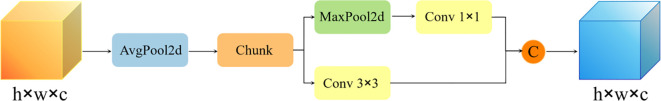
An Architecture of the proposed ADown module.

In **Figure 2**, c denotes the number of output channels after feature concatenation and channel compression.

For clarity, the pooling, concatenation, and convolution operations in this subsection are described in textual form, whereas the symbol ⊙ is reserved for element-wise multiplication in the attention modules.

### 2.3 Simple, Parameter-Free Attention Module

Attention mechanisms have been widely applied in object detection networks to enhance feature discriminability by strengthening key informative regions and suppressing irrelevant background responses. Most existing attention modules rely on additional convolutional layers or learnable parameters, which inevitably increase computational complexity and limit their application in lightweight detection frameworks. In agricultural pest and disease detection tasks, models are often required to balance detection accuracy with real-time performance, thereby imposing higher demands on computational efficiency and deployment friendliness.

To address these challenges, this study introduces a parameter-free attention mechanism, namely the Simple, Parameter-Free Attention Module (SimAM) (Yang et al., 2021), into the YOLO detection framework. The module enhances feature representations without introducing any additional learnable parameters, making it particularly suitable for lightweight object detection models.

The core concept of SimAM is derived from energy-function-based modeling, where attention weights are assigned by evaluating the discriminative contribution of individual neurons in feature representation. Let the input feature map be defined as:


$$\text{F}\in {\mathbb{R}}^{\text{H}\times \text{W}\times \text{C}}$$

SimAM measures the importance of each neuron by computing its deviation from other neurons within the same channel. Specifically, the energy value $\text{E}({\text{f}}_{\text{i}})$ of neuron ${\text{f}}_{\text{i}}$ is defined as:


$$\text{E}({\text{f}}_{\text{i}})=\frac{{\left({\text{f}}_{\text{i}}-\text{μ}\right)}^{2}}{{\text{σ}}^{2}+\text{λ}}$$

where f_i_ denotes the i-th neuron in a given channel, μ and ${\text{σ}}^{2}$ denote the mean and variance of the neurons within the same channel, respectively, and λ is a small regularization constant introduced to ensure numerical stability. $\text{E}({\text{f}}_{\text{i}})$ denotes the energy value of neuron ${\text{f}}_{\text{i}}$, where a lower value indicates stronger discriminative importance.

After obtaining the energy values for all neurons, SimAM applies a Sigmoid function to generate normalized attention weights, which are then multiplied element-wise with the original feature map to produce the enhanced representation:


$$\text{F}\prime =\text{F}⊙\text{σ}(\frac{1}{\text{E}(\text{F})})$$

where ⊙ denotes element-wise multiplication. This process adaptively emphasizes salient regions while effectively suppressing background noise.

In the proposed model, SimAM is integrated into the Neck stage and primarily applied to the intermediate-scale feature maps at the P3 and P4 levels. These layers retain relatively high spatial resolution while gradually incorporating semantic information, making them susceptible to interference from complex leaf textures and greenhouse background structures. By introducing SimAM at this stage, the model can more accurately distinguish insect bodies from lesion regions, thereby improving detection performance for small-scale pests and low-contrast disease features.

**Figure 3:**
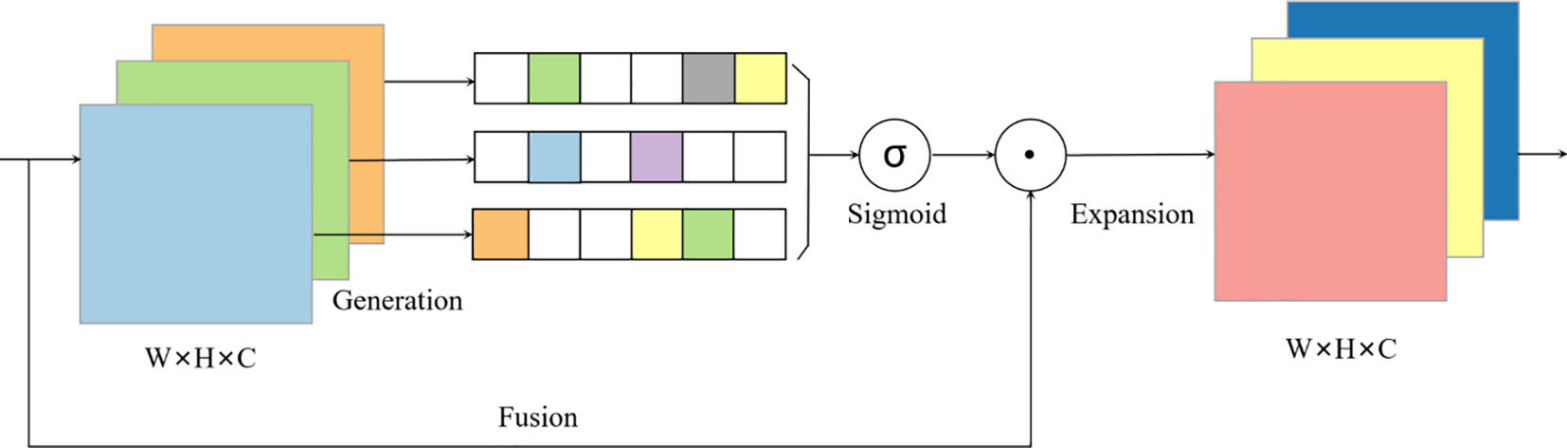
An Architecture of the proposed SimAM module.

Since SimAM does not introduce additional parameters or convolution operations, its impact on model size and computational cost is negligible. With SimAM incorporated, feature responses become more stable, and detection accuracy is improved for visually similar disease categories as well as small-scale pest targets. The architecture of the proposed SimAM module is illustrated in **Figure 3**.

### 2.4 Large Selective Kernel Attention

In desert greenhouse crop pest and disease detection scenarios, targets often exhibit substantial variations in spatial scale and morphological structure. Disease regions may spread extensively across leaf surfaces, whereas insect targets are frequently occluded by dense foliage or irrigation facilities. These characteristics impose higher requirements on the network’s ability to capture long-range contextual information and maintain semantic consistency under complex backgrounds. Traditional convolution operations based on small kernels have limited receptive fields and are insufficient for effectively modeling large-scale spatial dependencies.

To address this issue, this study introduces the Large Selective Kernel Attention (LSKAttention) module (Li et al., 2023) into the proposed YOLO detection framework to enhance high-level semantic feature representation. LSKAttention constructs large receptive fields combined with a selective fusion mechanism, enabling adaptive enhancement of key informative regions and improving robustness to scale variation and background interference.

The core structure of LSKAttention consists of multi-branch depthwise separable convolutions, where different branches employ convolution kernels of varying sizes to model multi-scale receptive fields. Let the input feature map be defined as:


$$\text{F}\in {\mathbb{R}}^{\text{H}\times \text{W}\times \text{C}}$$

Features are extracted through two parallel depthwise convolution branches:


$${\text{F}}_{1}={\text{DWConv}}_{{\text{k}}_{1}}(\text{F}),\;{\text{F}}_{2}={\text{DWConv}}_{{\text{k}}_{2}}(\text{F})$$

where ${\text{k}}_{1}$ and ${\text{k}}_{2}$ represent smaller and larger kernel sizes, respectively. This parallel structure enables the network to simultaneously capture local texture details and broader contextual information.

The multi-scale features are then aggregated to obtain a fused representation:


$${\text{F}}_{\text{sum}}={\text{F}}_{1}+{\text{F}}_{2}$$

A spatial attention map is generated via pointwise convolution and a Sigmoid activation function:


$${\text{M}}_{\text{LSK}}=\text{σ}({\text{Conv}}_{1\times 1}({\text{F}}_{\text{sum}}))$$

Finally, the enhanced feature representation is obtained through element-wise weighting:


$$\text{F}\prime =\text{F}⊙{\text{M}}_{\text{LSK}}$$

where ⊙ denotes element-wise multiplication. Through this selective kernel attention mechanism, the model emphasizes spatial regions that contribute more significantly to target discrimination while effectively suppressing irrelevant background responses.

In the proposed model, LSKAttention is deployed at the P5 feature level within the Neck stage. The P5 layer possesses lower spatial resolution but stronger semantic representation capability. Introducing large receptive field attention at this scale enhances semantic correlation across spatial regions, thereby improving the modeling of large-area lesion regions and insect damage patterns characterized by continuous structural features. This further enhances localization stability and semantic consistency during multi-form target detection.

**Figure 4:**
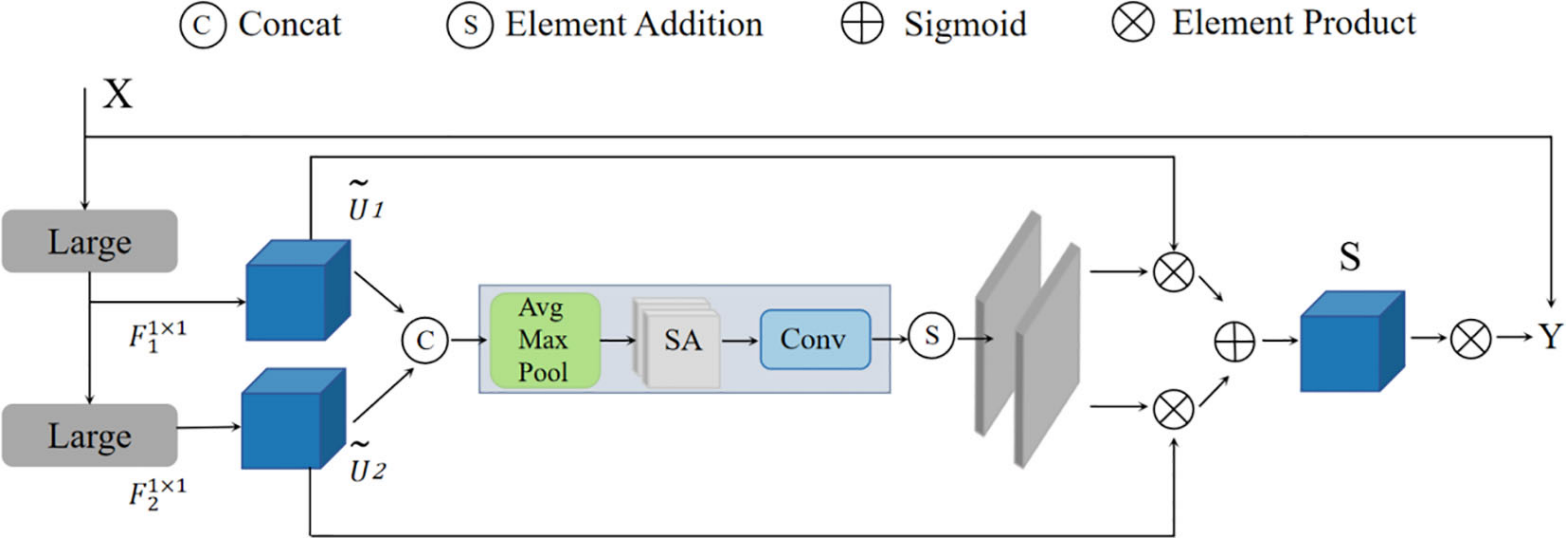
An Architecture of the proposed LSKAttention module.

LSKAttention employs depthwise and pointwise convolutions to construct a selective large-kernel attention mechanism, thereby controlling parameter scale and reducing computational complexity while avoiding highly redundant parameterized structures. Compared with directly stacking large convolution kernels, this design significantly reduces parameters and FLOPs while effectively expanding the receptive field. Consequently, a balanced trade-off is achieved between detection accuracy improvement and computational overhead, making the model more suitable for real-time pest and disease detection in desert greenhouse environments. The architecture of the proposed LSKAttention module is illustrated in **Figure 4**.

### 2.5 Dataset

The dataset constructed in this study is based on publicly available datasets and supplemented with images collected through Google and Bing search engines. During image acquisition, copyright regulations were strictly observed. Only images from webpages explicitly authorized for academic research or non-commercial use, and without restrictive copyright statements, were included. For webpages with unclear licensing information, only the corresponding URLs were retained, and the raw images were not incorporated into the dataset.

Initial bounding boxes and category labels were produced using the LabelImg tool by three professionally trained annotators. To ensure annotation reliability, approximately 15% of the annotated samples were independently reviewed by two plant protection specialists, and any discrepancies were resolved through consensus discussion. For annotation consistency, each visible lesion or pest instance was labeled with an individual bounding box. When multiple lesions or multiple instances appeared on the same leaf, they were annotated separately rather than merged into a single box. Healthy leaves and diseased leaves were assigned according to their predefined category labels in the dataset. Annotation files were generated in XML format following the PASCAL VOC 2007 standard, recording image names, image sizes, target categories, and bounding-box locations.

The dataset covers nine major categories of common greenhouse cash crops, including cucumber, eggplant, potato, and tomato, encompassing nine healthy leaf conditions and 29 disease and pest categories. To ensure data quality and eliminate redundancy, perceptual hashing techniques were employed to detect duplicate or near-duplicate images both within the dataset and among web-collected samples. All automatically identified duplicates were manually reviewed by two researchers. Additionally, images with low resolution, severe blur, overexposure, large-area occlusion, or prominent watermarks were removed. Ultimately, a high-quality dataset containing 38,371 images was constructed, with all images standardized to a resolution of 640 × 640 pixels. The dataset was subsequently divided into a training set and a validation set at a ratio of 9:1 to support model development and performance evaluation. Detailed category definitions and sample quantities are presented in **Table 1**.

**Table 1:** Dataset category details.

Species	Class	Num	Category
Bean	Healthy Leaf	1093	Healthy Plant
	Angular Leaf Spot	1366	Fungal Disease
	Anthracnose	899	Fungal Disease
	Blight	894	Fungal Disease
	Rust	895	Fungal Disease
	Mosaic Virus	903	Viral Disease
Bell Pepper	Healthy Leaf	1309	Healthy Plant
	Leaf Spot	998	Fungal Disease
Bottle Gourd	Healthy Leaf	1250	Healthy Plant
	Mosaic Virus	1017	Viral Disease
Cauliflower	Healthy Leaf	957	Healthy Plant
	Downy Mildew	977	Fungal Disease
	Black Rot	886	Bacterial Disease
Cucumber	Healthy Leaf	899	Healthy Plant
	Downy Mildew	790	Fungal Disease
Eggplant	Healthy Leaf	1041	Healthy Plant
	Leaf Mosaic	939	Viral Disease
	Powdery Mildew	921	Fungal Disease
Peas	Healthy Leaf	1017	Healthy Plant
	Downy Mildew	907	Fungal Disease
	Leaf Miner	921	Insect Pest
	Powdery Mildew	893	Fungal Disease
	Ascochyta Blight	537	Fungal Disease
Potato	Healthy Leaf	1161	Healthy Plant
	Early Blight	1013	Fungal Disease
	Late Blight	1142	Fungal Disease
Tomato	Healthy Leaf	1153	Healthy Plant
	Spider Mites	907	Insect Pest
	Bacterial Spot	1057	Bacterial Disease
	Brown Spot	1260	Fungal Disease
	Early Blight	943	Fungal Disease
	Late Blight	881	Fungal Disease
	Leaf Curling	931	Viral Disease
	Leaf Mold	936	Fungal Disease
	Mosaic Virus	1070	Viral Disease
	Powdery Mildew	1022	Fungal Disease
	Septoria Leaf Spot	1484	Fungal Disease
	Yellow Virus	1102	Viral Disease

To ensure domain consistency, web-collected images were manually screened to retain only samples exhibiting greenhouse cultivation characteristics consistent with desert greenhouse conditions. Images captured under open-field environments were excluded. Furthermore, all images were re-annotated and verified by plant protection specialists to ensure biological and contextual correctness. The dataset will be made available for academic research upon reasonable request, subject to licensing constraints.

Although efforts were made to ensure domain consistency, the dataset may still exhibit distribution differences compared with real-world desert greenhouse environments. The independent test set was therefore introduced to further evaluate the real-world generalization capability of the proposed model under practical desert greenhouse conditions.

### 2.6 Experimental environment

The proposed YOLO-based object detection model was trained for 100 epochs with a batch size of 64. All input images were uniformly resized to a fixed resolution of 640 × 640 to achieve a balance between detection accuracy and computational efficiency. Considering the specific characteristics of desert greenhouse environments—such as intense and uneven illumination, strong reflections caused by greenhouse films, background interference from irrigation facilities, and local occlusions between crop leaves—the default data augmentation strategies provided by the YOLOv11s framework were adopted during training, including Mosaic augmentation, random horizontal flipping, HSV color-space augmentation, scaling, and translation, with parameter settings following the official implementation, to improve model robustness and generalization under complex visual conditions.

All experiments were conducted on a workstation running Ubuntu 22.04. The implementation was based on Python 3.9 and the PyTorch 2.9.0 deep learning framework. In terms of hardware configuration, an Intel Xeon Platinum 8470 Q CPU was used, with 90 GB of system memory. The GPU was an NVIDIA RTX 5090 with 32 GB of video memory, and CUDA 12.8 was employed to accelerate both training and inference. The optimizer was SGD with a momentum of 0.937, an initial learning rate of 0.01, a cosine annealing learning rate scheduler with a three-epoch warm-up, and a weight decay of 0.0005.

Pretrained weights were adopted to initialize all detection models in order to accelerate convergence and improve optimization stability. To ensure experimental fairness, all compared models were trained under identical initialization settings, input resolution, batch size, optimizer configuration, and training schedules. No additional external datasets were used for task-specific fine-tuning, ensuring that performance comparisons reflect architectural differences rather than training advantages.

Unless otherwise specified, the default augmentation and post-processing settings provided by the official YOLOv11s implementation were adopted. During evaluation, all models were tested under the same confidence threshold and non-maximum suppression settings.

### 2.7 Evaluation metrics

To comprehensively evaluate the detection performance of the proposed model for crop pest and disease detection in desert greenhouse scenarios, multiple commonly used metrics in the object detection field were adopted, including Precision, Recall, mean Average Precision (mAP), Parameters, floating-point operations (FLOPs), and inference speed measured in Frames Per Second (FPS) (Yang et al., 2025). Among these, Precision, Recall, and mAP mainly quantify detection accuracy and stability, whereas Parameters, FLOPs, and FPS are used to assess computational overhead and real-time performance.

Precision represents the proportion of true positives among all predicted positive detections, and is computed as:


$$\text{Precision}=\frac{\text{TP}}{\text{TP}+\text{FP}}$$

where TP denotes the number of correctly detected targets, and FP denotes the number of falsely detected targets.

Recall measures the coverage of real targets by the model, defined as:


$$\text{Recall}=\frac{\text{TP}}{\text{TP}+\text{FN}}$$

where FN denotes the number of real targets missed by the detector.

To reflect detection performance across different categories, Average Precision (AP) was used as the single-class evaluation metric. AP is obtained by integrating the Precision–Recall curve. Furthermore, the mean Average Precision (mAP) is computed by averaging AP over all categories, defined as:


$$mAP=\frac{1}{N}\sum_{i=1}^{N}AP_{i}$$

where N is the total number of target categories and AP_i_ denotes the average precision of the i-th category.

Intersection over Union (IoU) is used to measure the overlap between the predicted bounding box and the ground-truth bounding box. It is calculated as the intersection area divided by the union area. When the IoU value reaches or exceeds 50%, the prediction is regarded as correct.

In the experiments, two criteria, mAP@0.5 and mAP@0.5–0.95, were employed. Specifically, mAP@0.5 refers to mAP calculated under an IoU threshold of 0.5, whereas mAP@0.5–0.95 is obtained by averaging AP over multiple IoU thresholds ranging from 0.5 to 0.95 with a step size of 0.05. This metric provides a stricter evaluation of localization accuracy and overall detection performance.

In addition, to assess feasibility in practical deployment scenarios, the number of parameters (Params) and FLOPs were further reported to reflect spatial complexity and computational complexity. Meanwhile, inference speed (FPS) was measured under a unified hardware setting to comprehensively evaluate runtime efficiency for real-time pest and disease detection applications in desert greenhouses.

## 3 Results and discussion

### 3.1 Ablation study

To systematically evaluate the effectiveness of the proposed modules within the YOLOv11 framework and their collaborative effects, a series of ablation experiments were designed and conducted. The original YOLOv11 detection model without any additional modules was selected as the baseline. On this basis, ADown, SimAM, and LSKAttention were successively incorporated into the network architecture. Under identical training settings and with the overall network topology unchanged, multiple model variants with different module combinations were constructed and comparatively analyzed.

The evaluation focused on core object detection metrics and lightweight characteristics, including Precision, Recall, mean Average Precision (mAP@0.5–0.95), FLOPs, and parameter count. Quantitative comparisons among model variants were performed to assess the individual contributions of ADown (A), SimAM (B), and LSKAttention (C) in terms of detection accuracy and computational efficiency, as well as the overall performance gains brought by their collaborative integration. The results indicate that combining ADown, SimAM, and LSKAttention improves detection accuracy while maintaining comparable computational complexity under the tested conditions.

**Figure 5:**
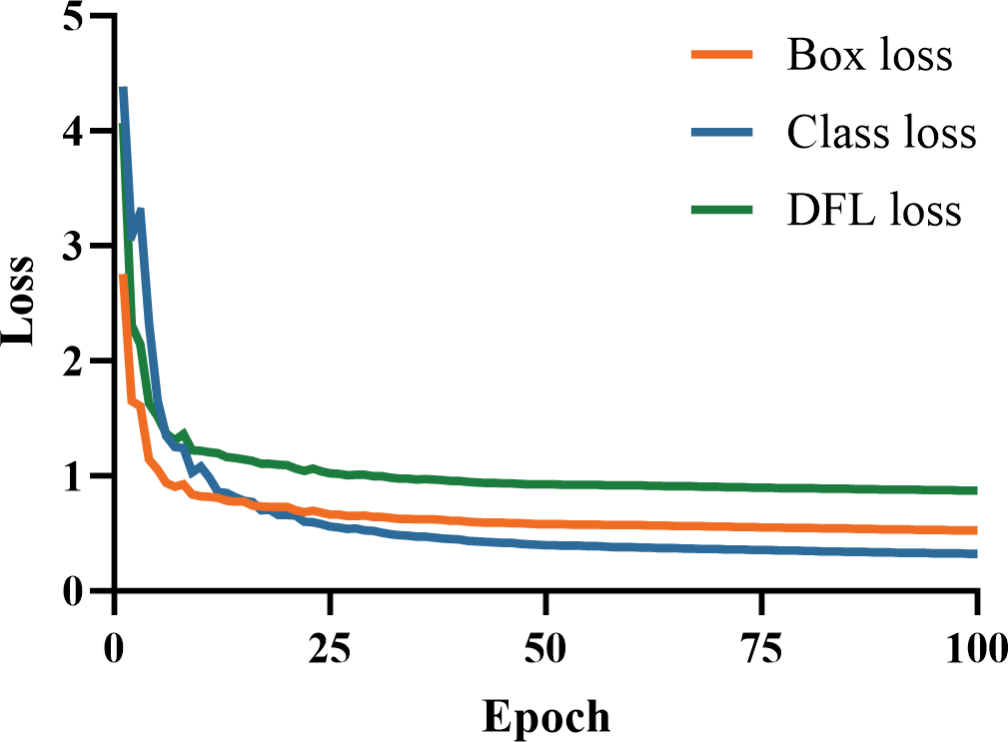
Training loss curve.

As shown in the training loss curves in **Figure 5**, the variation trends of each loss component during training are illustrated, where the horizontal axis denotes epochs and the vertical axis denotes loss values. As observed, Box Loss, Class Loss, and DFL Loss decrease rapidly at the early training stage and gradually stabilize as training proceeds.

Specifically, Class Loss exhibits the largest decrease, with a relatively high initial value but a rapid reduction within the first several epochs, indicating that the classification branch quickly enters an effective learning stage. Box Loss also declines noticeably at the beginning and then decreases more slowly, reflecting a gradual improvement in localization accuracy. DFL Loss becomes relatively stable after an initial decline, suggesting that the distribution regression branch maintains steady optimization during the mid-to-late training stage. Overall, the loss curves are smooth with a clear convergence trend, indicating good training stability and convergence behavior.

The ablation results are summarized in **Table 2**. Compared with the baseline model, the proposed model integrating all three modules (A+B+C) achieves the best overall performance across all evaluation metrics. Specifically, mAP@0.5–0.95 improves from 0.826 to 0.860, representing an absolute gain of 3.4 percentage points. Meanwhile, Precision and Recall increase to 95.7% and 93.1%, respectively, indicating enhanced detection reliability and robustness. In addition, mAP@0.5 reaches 0.968, further demonstrating superior detection performance under a moderate IoU threshold.

**Table 2:** Ablation results.

Model	Layers	Params(M)	GFLOPs	P	R	mAP@0.5	mAP@0.5-0.95	size(MB)	FPS
baseline	100	9.43	21.4	0.923	0.902	0.952	0.826	18.3	96
A	112	7.90	17.5	0.932	0.914	0.957	0.838	15.4	122
B	100	9.43	21.6	0.940	0.918	0.960	0.842	18.4	93
C	112	10.49	22.8	0.944	0.922	0.964	0.852	20.3	88
A+B	112	7.90	17.7	0.945	0.922	0.962	0.850	15.5	119
A+C	118	8.14	17.8	0.948	0.925	0.966	0.856	15.8	113
B+C	112	10.49	23.0	0.949	0.926	0.967	0.857	20.4	91
A+B+C	118	8.14	17.9	0.957	0.931	0.968	0.860	15.9	116

In the single-module experiments, the ADown module (A) shows clear advantages in balancing lightweight design and detection accuracy. Compared with the baseline, it reduces the number of parameters from 9.43 M to 7.90 M and decreases computational complexity from 21.4 GFLOPs to 17.5 GFLOPs, while improving mAP@0.5–0.95 from 0.826 to 0.838. This result suggests that optimizing the down-sampling process contributes positively to feature preservation in crop pest and disease detection tasks. Similar findings were reported by Zhang et al (Zhang and Jiang, 2025), who observed that incorporating ADown into YOLOv11n reduced model parameters while improving detection accuracy, supporting its effectiveness in lightweight model design.

The LSKAttention module (C) also provides notable performance improvements. By enlarging the receptive field and introducing a selective attention mechanism, it enhances feature representation under complex background conditions. As shown in **Table 2**, incorporating LSKAttention alone increases mAP@0.5–0.95 to 0.852, outperforming both the baseline and other single-module configurations.

In contrast, the performance gain brought by SimAM (B) alone is relatively moderate, with mAP@0.5–0.95 improving to 0.842. Nevertheless, it still contributes to performance enhancement through adaptive feature recalibration. Previous studies have similarly reported consistent improvements after integrating SimAM into YOLO-based frameworks for plant disease detection (Liu et al., 2025), indicating its effectiveness in suppressing background interference and strengthening lesion-focused feature representation. In this study, although its standalone improvement is limited, its integration with other modules further enhances overall performance.

Further analysis of two-module combinations reveals complementary effects among modules. Configurations including LSKAttention (A+C and B+C) achieve higher mAP@0.5–0.95 scores (0.856 and 0.857, respectively) than the A+B combination (0.850), suggesting that the large-receptive-field attention mechanism contributes positively to multi-module synergy. When all three modules are integrated, the model attains the best comprehensive performance, indicating that their joint optimization effectively strengthens feature extraction and representation capability.

Moreover, while maintaining high detection accuracy, the final model contains only 8.14 M parameters and 17.9 GFLOPs, with a model size of 15.9 MB. The inference speed reaches 116 FPS, significantly higher than the baseline (96 FPS), demonstrating its potential suitability for real-time crop pest and disease detection in desert greenhouse environments.

The superior performance of the full model further supports the rationality of the hierarchical deployment strategy. ADown mainly contributes to robust multi-scale construction under resolution changes, SimAM enhances intermediate feature discrimination for small targets, and LSKAttention strengthens high-level semantic aggregation. Their complementary effects explain why the A+B+C configuration achieves the best balance between accuracy and efficiency.

### 3.2 Comparison between baseline YOLOv11s and YOLOv11s-DesertAttn

**Figure 6:**
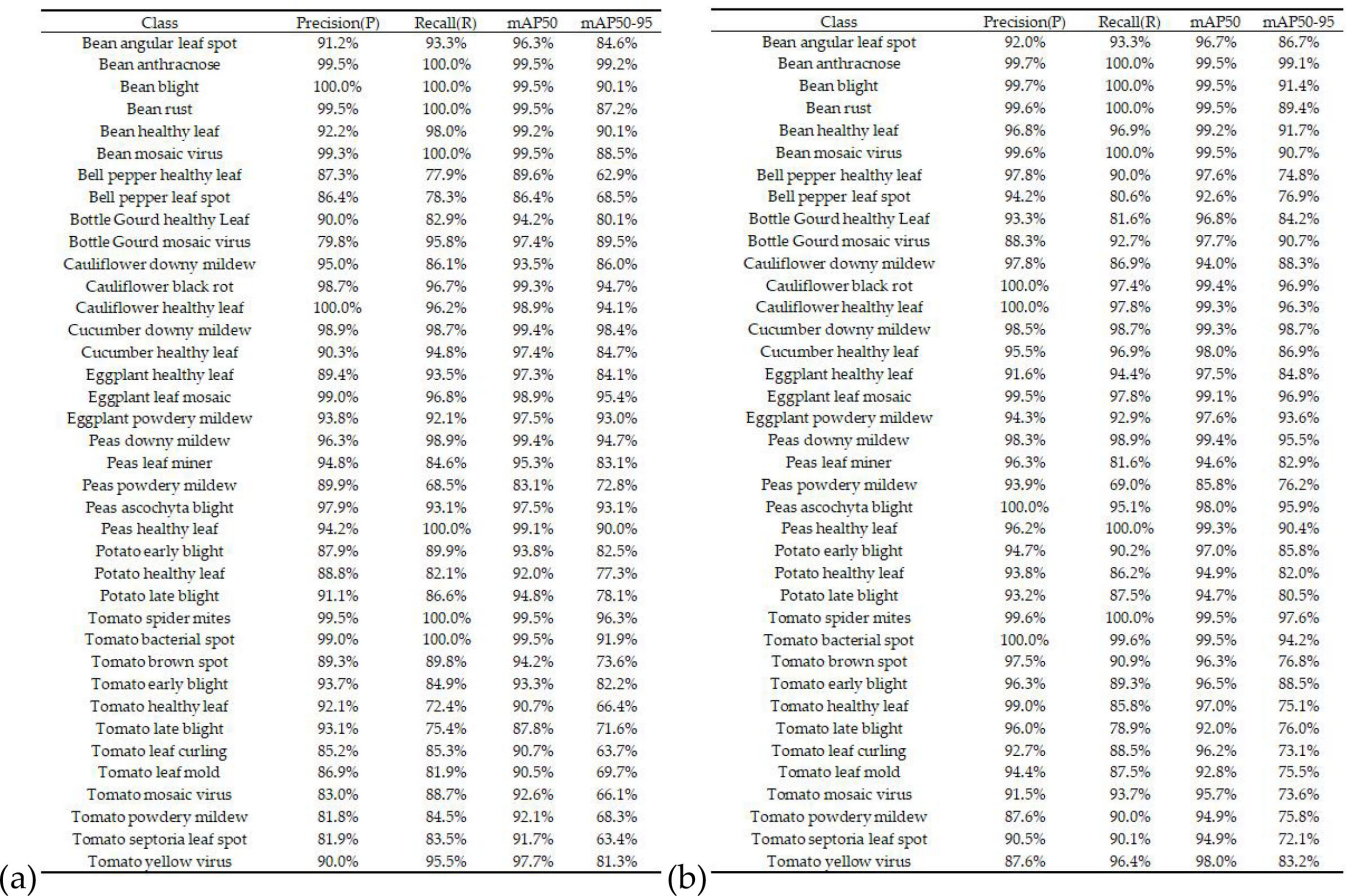
Comparison chart of mAP@0.5–0.95; **(a)** YOLOv11s; **(b)** YOLOv11s-DesertAttn.

To visually assess the effectiveness of the proposed improvement strategy, the detection performance of the baseline model YOLOv11s and the improved model YOLOv11s-DesertAttn was compared on the same test set, as shown in **Figure 6**.

**Figure 7:**
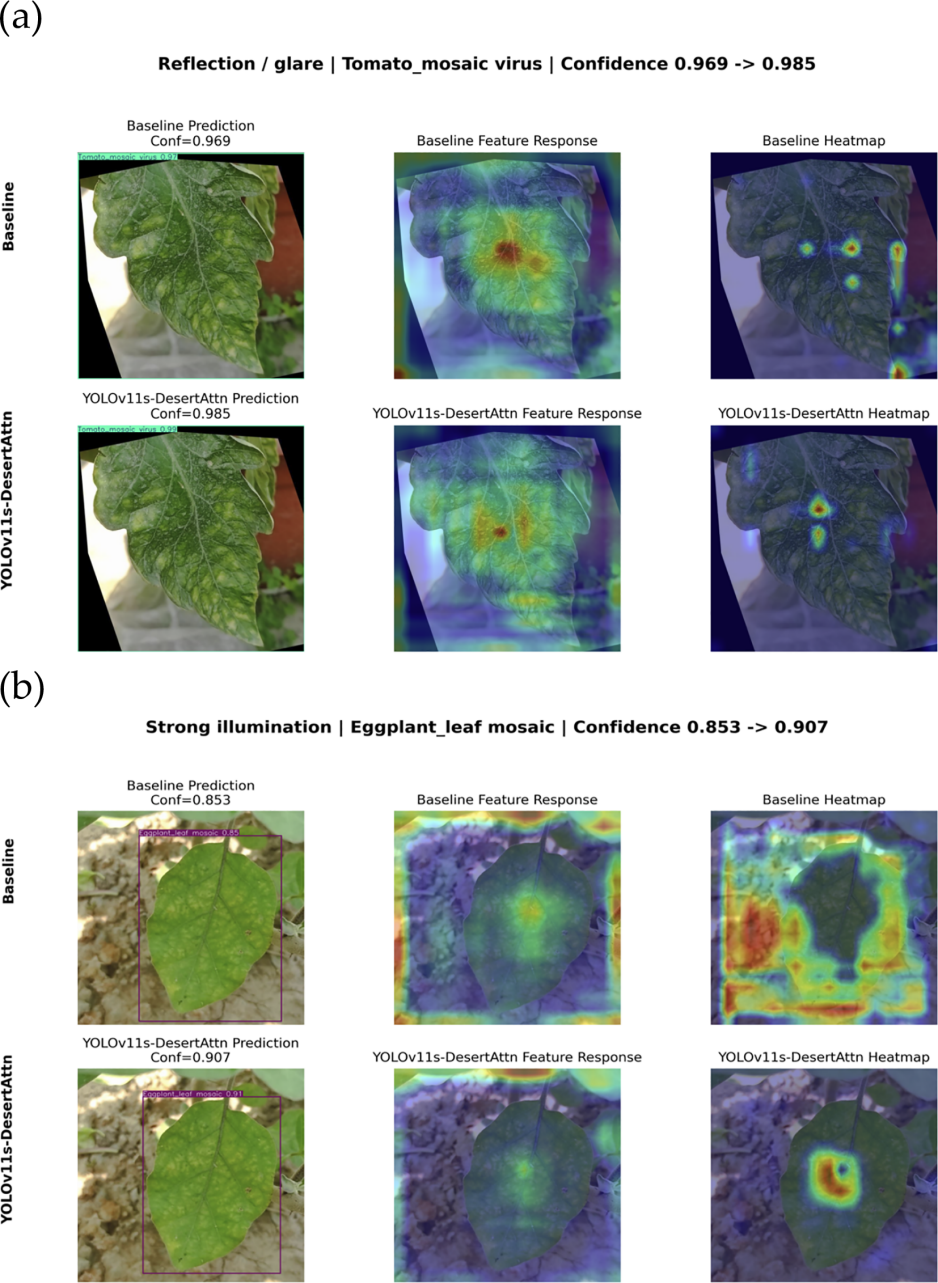
Visual comparison of feature responses and attention heatmaps between the baseline and YOLOv11s-DesertAttn under **(a)** strong illumination and **(b)** reflective interference conditions.

To further support the effectiveness of the proposed method under strong illumination, reflective interference, and partial occlusion, additional visual comparisons including feature-response maps and attention heatmaps are presented in **Figure 7**. These visualizations illustrate how the model focuses on disease-relevant regions while suppressing background noise.

In desert greenhouse crop pest and disease detection scenarios, the comparison in **Figure 6** reveals substantial differences between the baseline YOLOv11s and YOLOv11s-DesertAttn. Under conditions such as leaf overlap, uneven illumination, or mixed occurrence of multiple diseases, the baseline model is prone to background false activations, missed detections of small targets, and category confusion. In contrast, YOLOv11s-DesertAttn systematically improves feature representation and localization accuracy by introducing anti-aliasing down-sampling (ADown), parameter-free attention (SimAM), and large receptive field attention (LSKAttention).

As indicated by the per-class metric comparisons in **Figure 6**, the improved model achieves clear gains on several easily confused disease categories: the mAP50–95 for tomato early blight increases from 82.2% to 88.5% (+6.3 percentage points), tomato leaf mold increases from 69.7% to 75.5% (+5.8 percentage points), tomato bacterial spot increases from 91.9% to 94.2% (+2.3 percentage points), and potato early blight increases from 82.5% to 85.8% (+3.3 percentage points). For small-scale pest targets, the mAP50–95 for tomato red spider mite reaches 97.6%, improving by 1.3 percentage points over the baseline; meanwhile, the mAP50–95 for cowpea angular leaf spot increases by 2.1 percentage points to 86.7%. Notably, under low-contrast conditions, weak textures, and partial occlusion, the improved model still maintains higher recall and confidence, demonstrating stronger adaptability and robustness.

These results are consistent with the trend reported by Xiong et al. (2025) (Xiong et al., 2025), indicating that a collaborative design combining lightweight convolutions with hierarchical attention mechanisms can effectively improve small-target disease detection in complex agricultural scenarios. Moreover, under harsher desert greenhouse conditions with finer-grained category definitions and more challenging illumination–background interference, the proposed YOLOv11s-DesertAttn achieves superior detection performance compared with baseline approaches, further validating the generality and advancement of the hierarchical fusion strategy for agricultural vision tasks in extreme environments.

Overall, YOLOv11s-DesertAttn significantly outperforms the baseline model in detection accuracy, anti-interference capability, and lightweight characteristics, providing solid technical support for real-time intelligent monitoring of crop pests and diseases in desert greenhouse environments. As shown in **Figure 7**, the proposed model produces more concentrated feature activations and attention responses on target regions, while effectively suppressing background interference caused by strong illumination and reflections.

### 3.3 Comparative experiment

To comprehensively evaluate the performance of the proposed model for multi-class pest and disease detection in desert greenhouse environments, YOLOv11s-DesertAttn was systematically compared with several representative object detection models, including classical YOLO-based detectors (YOLOv5s, YOLOv7-tiny, and YOLOv8s), the anchor-free detector YOLOX-s, the transformer-based detector RT-DETR-R18, the lightweight mobile-oriented model SSDLite-MobileNetV3-Large, Faster R-CNN (R50-FPN), and the recently proposed RTMDet-s. All models were trained and evaluated on the same dataset under identical experimental settings to ensure fairness and comparability.

As shown in **Table 3**, YOLOv11s-DesertAttn achieves an excellent balance between detection accuracy and computational efficiency. Specifically, the proposed model attains an mAP@0.5–0.95 of 86.0%, which is significantly higher than that of YOLOv5s (72.3%) (Liu et al., 2020), YOLOv7-tiny (75.1%), YOLOX-s (78.3%), and YOLOv8s (81.4%) (Wang et al., 2023), and also surpasses Faster R-CNN (R50-FPN) (80.3%) (Byenkya et al., 2026). These results demonstrate the superior capability of the proposed model in detecting multi-scale pest and disease targets under complex greenhouse conditions.

**Table 3:** Comparison of results of different target detection models.

Model	Params (M)	GFLOPs	R/%	mAP@0.5/%	mAP@0.5-0.95/%	size(MB)	FPS
YOLOv11s baseline	9.43	21.4	90.2	95.2	82.6	18.3	96
YOLOv5s	7.11	16.1	82.1	88.3	72.3	13.9	135
YOLOv7-tiny	6.14	13.8	83.2	89.5	75.1	12.2	145
YOLOv8s	11.14	28.5	89.3	94.5	81.4	21.5	73
YOLOX-s	8.93	26.5	84.9	91.8	78.3	35.8	65
RT-DETR-R18	20.12	60.3	92.2	96.3	85.4	77.5	31
Ssd-MobileNetV3 large	2.72	2.3	77.2	80.7	67.5	10.6	268
Faster R-CNN(R50-FPN)	41.63	181.5	86.8	93.2	80.3	159.1	10
RTMDet-s	8.87	14.8	89.5	94.9	81.7	34.2	138
YOLOv11s-DesertAttn	8.14	17.9	93.1	96.8	86.0	15.9	116

(a)YOLOv5s(b)YOLOv8s(c)YOLOv11s_baseline(d)YOLOv11s-DesertAttn.

Compared with the original YOLOv11s baseline, YOLOv11s-DesertAttn improves mAP@0.5–0.95 from 82.6% to 86.0% and increases Recall from 90.2% to 93.1%. Meanwhile, the number of parameters is reduced from 9.43 M to 8.14 M, and the computational complexity decreases from 21.4 GFLOPs to 17.9 GFLOPs. Inference speed is also improved from 96 FPS to 116 FPS. These improvements indicate that the proposed information-preserving down-sampling strategy, intermediate lightweight attention module, and high-level large receptive field attention mechanism effectively enhance feature extraction and representation capability while simultaneously reducing computational overhead.

Among lightweight detectors, YOLOv7-tiny achieves relatively high inference speed (145 FPS) with a compact parameter scale of 6.14 M; however, its mAP@0.5–0.95 reaches only 75.1%, which is still considerably lower than that of the proposed model. Similarly, YOLOX-s achieves 78.3% mAP@0.5–0.95, but requires higher computational complexity (26.5 GFLOPs) and a larger model size (35.8 MB), while still failing to achieve comparable detection accuracy. These results indicate that simply reducing model size or adopting lightweight structures cannot effectively guarantee robust feature representation for fine-grained pest and disease detection tasks.

Among the lightweight models, SSDLite-MobileNetV3-Large achieves the highest inference speed (268 FPS) with only 2.72 M parameters; however, its detection accuracy remains relatively low, with an mAP@0.5–0.95 of only 67.5%. Such performance is insufficient for practical fine-grained multi-class pest and disease detection tasks. In contrast, YOLOv11s-DesertAttn maintains high detection accuracy while still achieving real-time inference speed, demonstrating better suitability for practical deployment in desert greenhouse environments.

Recent studies have also highlighted the importance of lightweight model design for edge-device deployment. Efficient deep learning architectures can significantly reduce computational cost while maintaining reliable detection accuracy in real-world applications (Ishfaque et al., 2026). In addition, lightweight convolution-based models have shown strong adaptability in resource-constrained environments, especially under complex backgrounds and varying illumination conditions (Zhang et al., 2026). The design philosophy of the proposed model is consistent with these findings.

**Figure 8:**
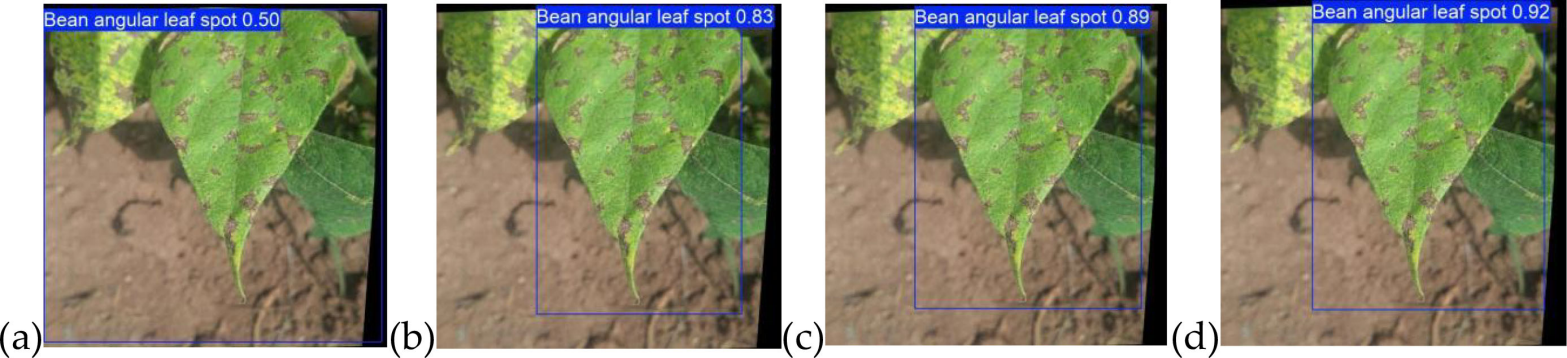
Comparison of visualization results from different models. **(a)** YOLOv5s **(b)** YOLOv8s **(c)** YOLOv11s_baseline **(d)** YOLOv11s-DesertAttn.

**Figure 8** presents visualization comparisons of different models on representative pest and disease samples. The proposed model exhibits more accurate localization and classification performance, particularly for small targets and densely distributed objects, further demonstrating the effectiveness of the proposed feature enhancement strategy.

In addition, RT-DETR-R18 achieves a comparable detection accuracy of 85.4% mAP@0.5–0.95. However, its parameter count (20.12 M) and computational complexity (60.3 GFLOPs) are substantially higher than those of YOLOv11s-DesertAttn, while its inference speed is only 31 FPS. These limitations reduce its practicality for deployment in resource-constrained agricultural environments. This comparison further verifies the advantage of the proposed model in balancing detection performance and computational efficiency.

Overall, YOLOv11s-DesertAttn achieves competitive detection accuracy while significantly reducing model complexity and maintaining superior inference speed compared with most existing methods. These results demonstrate the effectiveness and practical value of the proposed hierarchical feature fusion strategy for pest and disease detection in desert greenhouse scenarios.

Although inference speed was evaluated on a high-performance GPU platform to ensure standardized comparison, the proposed model remains lightweight, containing only 8.14 M parameters and requiring 17.9 GFLOPs. Such computational requirements are well within the deployment capability of mainstream embedded inference platforms, such as NVIDIA Jetson-series devices. Considering that real-time greenhouse monitoring systems typically require frame rates of only 15–30 FPS, the achieved inference speed of 116 FPS provides sufficient computational redundancy for edge deployment after optimization techniques such as model quantization or TensorRT acceleration. Therefore, the proposed model demonstrates strong potential for practical deployment in resource-constrained edge computing environments.

### 3.4 Per-class performance analysis

**Figure 9:**
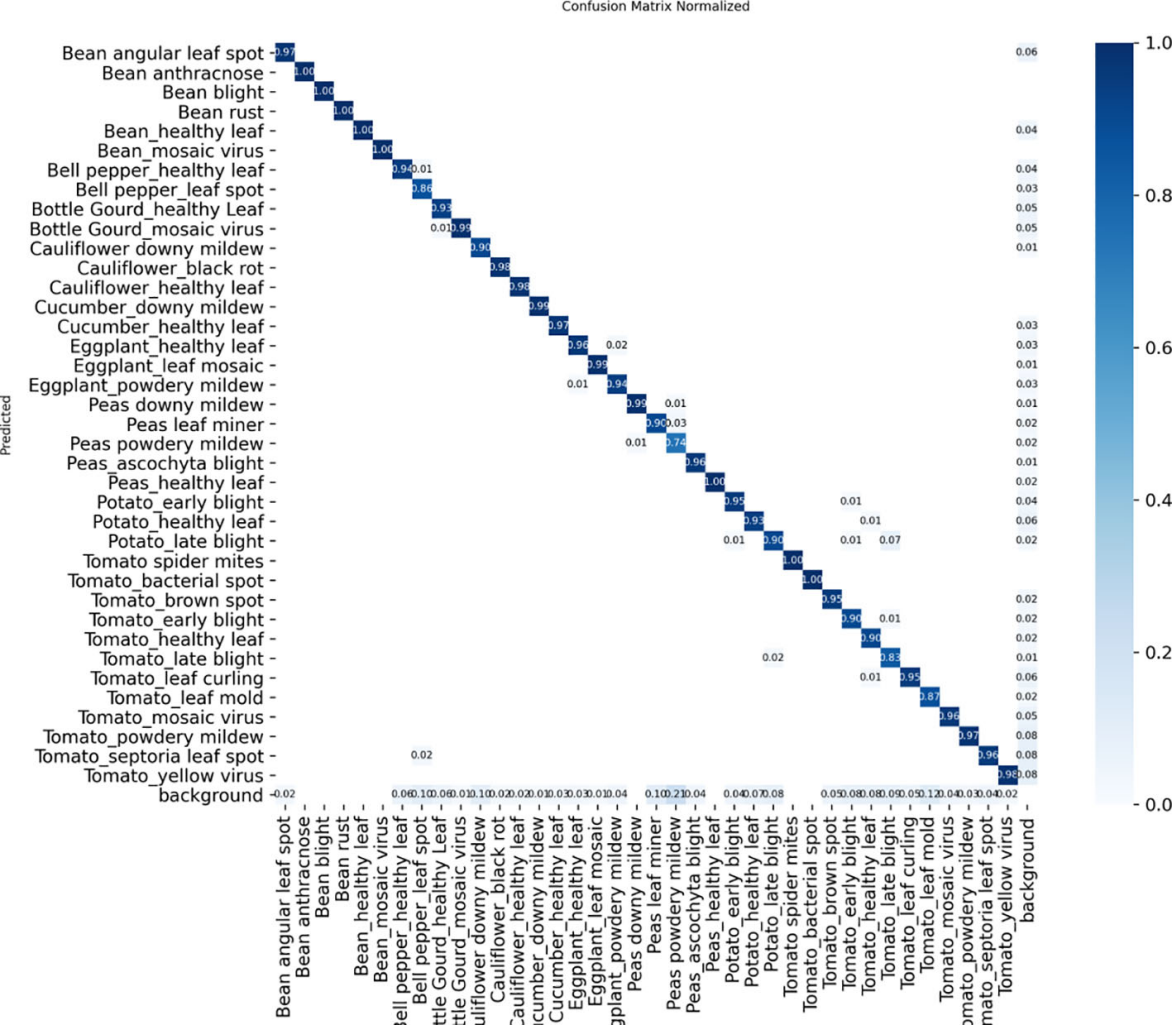
The confusion matrix (YOLOv11s-DesertAttn).

**Figure 10:**
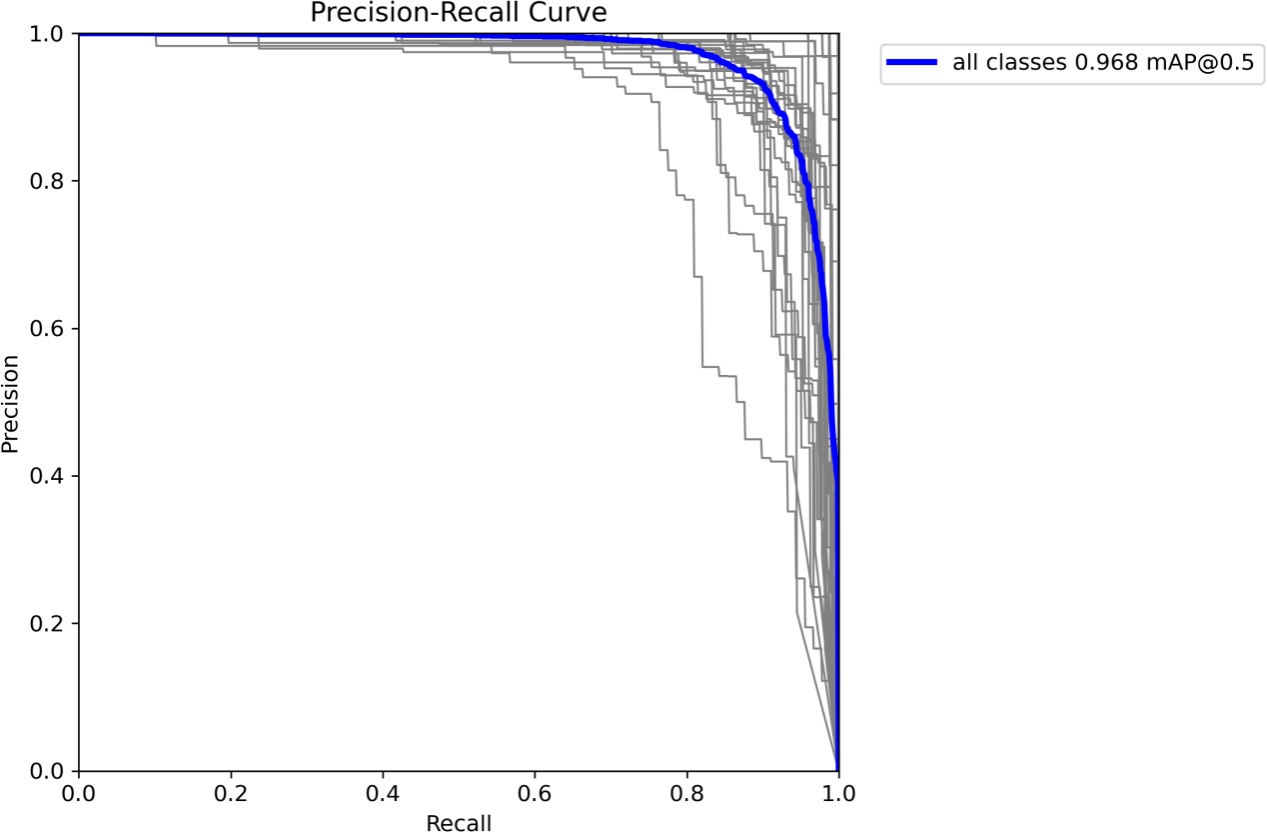
PR (YOLOv11s-DesertAttn).

To further evaluate class-level detection performance, the confusion matrix and Precision–Recall (PR) curves of the improved YOLOv11s-DesertAttn model are presented in **Figures 9** and **10**, respectively.

The confusion matrix shows that the true positive rates of most categories are above 0.95, and predictions are highly concentrated along the diagonal, indicating excellent class discriminability. Only a few diseases with highly similar visual features exhibit slight confusion, such as Tomato healthy leaf vs. Tomato yellow virus, Peas healthy leaf vs. Peas powdery mildew, Tomato early blight vs. Tomato brown spot, and Eggplant powdery mildew vs. Eggplant leaf mosaic. This is mainly due to overlapping early symptoms and similar textures, which is a common phenomenon in complex agricultural scenarios.

The PR curves further confirm model robustness. The overall mAP@0.5 reaches 96.8%, and the macro-average curve remains close to the upper boundary across a wide recall range. Categories such as cucumber downy mildew, cowpea anthracnose, tomato bacterial spot, and cauliflower black rot exhibit near “right-angle” PR curves, maintaining precision above 0.95 even when recall approaches 1.0. Only a small number of healthy categories, such as tomato healthy leaf and potato healthy leaf, show a slight precision decline at high recall, likely because of visual continuity with early asymptomatic infection samples.

By comparison, the model incorporating the improved down-sampling structure with feature enhancement and optimized feature fusion shows an obvious reduction in misclassifications for the above categories, particularly for small lesion targets and low-contrast disease categories, where diagonal responses are further strengthened. These results indicate that the improved model can more effectively preserve discriminative information related to pests and diseases during down-sampling and feature representation, thereby improving class-level discrimination accuracy.

Overall, the confusion matrix results visually validate the effectiveness of the proposed strategy in complex desert greenhouse pest and disease detection scenarios and provide structural evidence for subsequent quantitative performance evaluation.

### 3.5 Application evaluation based on an independent test set

To further evaluate cross-domain robustness and practical applicability, an independent test set containing 4,328 real-world images was constructed. All images were collected between March and June 2025 inside the greenhouses of a 10,000-mu facility agriculture base in Hotan, Xinjiang. Image acquisition covered diverse conditions, including natural illumination, supplemental artificial lighting, leaf occlusion, and complex backgrounds, in order to simulate realistic desert greenhouse environments.

All images were annotated following the same protocol as the main dataset. Initial bounding boxes and category labels were produced by three professionally trained annotators. Subsequently, two plant pathology experts independently reviewed approximately 600 stratified samples (about 14% of the dataset). Discrepancies were resolved through joint discussion until consensus was reached, ensuring annotation reliability and reducing potential systematic bias. The dataset therefore provides a rigorous evaluation of model performance under realistic greenhouse conditions.

Performance comparisons are summarized in **Table 4**. YOLOv11s-DesertAttn consistently outperforms the baseline model across all evaluation metrics. Specifically, the improved model achieves an mAP@0.5–0.95 of 85.6%, a Recall of 92.6%, a Precision of 94.9%, and an inference speed of 115 FPS, whereas the baseline attains 81.7% mAP@0.5–0.95, 88.6% Recall, 90.8% Precision, and 93 FPS. These results suggest that the collaborative integration of ADown, SimAM, and LSKAttention contributes to improved robustness under complex illumination, occlusion, and background interference.

**Table 4:** Performance comparison on the independent test set.

Model	Params (M)	GFLOPs	P/%	R/%	mAP@0.5/%	mAP@0.5–0.95/%	Size (MB)	FPS
baseline	9.43	21.4	90.8	88.6	94.4	81.7	18.3	93
YOLOv11s-DesertAttn	8.14	17.9	94.9	92.6	96.5	85.6	15.9	115

Meanwhile, the improved model reduces parameters from 9.43 M to 8.14 M and decreases computational complexity from 21.4 GFLOPs to 17.9 GFLOPs, while compressing model size from 18.3 MB to 15.9 MB. This indicates that enhanced detection performance is achieved alongside improved computational efficiency, supporting deployment on resource-constrained edge devices in desert greenhouse environments.

It is important to note that the main training dataset and the independent real-world test set differ in data source composition and visual distribution. This reflects an inherent domain gap between curated datasets and real-world desert greenhouse environments, particularly in terms of illumination conditions, background complexity, crop arrangement, and occlusion patterns.

Although the proposed model demonstrates strong performance on the independent test set, these results indicate that domain transfer remains a challenge in practical deployment. Future work will focus on region-specific data expansion, cross-domain validation, and domain adaptation strategies to further improve generalization performance.

## 4 Conclusions

1. By integrating the ADown down-sampling module, the SimAM parameter-free attention mechanism, and the LSKAttention large selective kernel attention module, a lightweight hierarchical feature-enhanced model architecture is constructed. While reducing computational complexity and parameter count, the proposed model significantly improves detection capability for small-scale and heavily occluded pest and disease targets under complex desert greenhouse conditions.
2. Systematic ablation experiments and comparative analyses confirm that the proposed model achieves a better trade-off between detection accuracy and inference efficiency. Compared with the original YOLOv11s baseline model and other mainstream detectors, YOLOv11s-DesertAttn demonstrates significantly better performance on key metrics such as mAP@0.5–0.95 and Recall, while further reducing parameter count and computational complexity, and increasing FPS to 116, indicating excellent overall performance.
3. The study clarifies the critical role of the hierarchical attention enhancement strategy in improving feature representation capability. The collaborative integration of SimAM and LSKAttention effectively enhances robustness under complex illumination, background interference, and leaf occlusion.
4. To further validate generalization performance and practical value, an independent test set containing 4328 real-world images collected from desert greenhouses in Hotan, Xinjiang was used for application evaluation. The improved model maintains excellent detection performance in unseen real scenarios, achieving an mAP@0.5–0.95 of 0.856, significantly outperforming the baseline model, thereby confirming strong cross-domain adaptability and deployment feasibility.

Nevertheless, several limitations remain. On the one hand, the crop categories and pest/disease types included in the dataset mainly target common greenhouse cash crops; the model’s generalization ability to a broader range of crops and rare diseases still requires further verification. In addition, potential domain differences between the constructed dataset and real-world greenhouse environments may affect generalization performance. On the other hand, this study focuses on performance optimization of visual detection algorithms and has not yet achieved deep data closed-loop integration and decision linkage with greenhouse environmental control systems.

Therefore, future work will proceed in three directions: (i) continuously expanding the dataset by incorporating more typical desert greenhouse crop species, special growth states, and extreme-environment samples to improve generalization across regions and production modes (Guo et al., 2024); (ii) exploring multimodal information fusion methods, such as integrating hyperspectral imaging, proximal environmental sensors, and crop physiological monitoring data, to build cross-modal collaborative perception models and improve pest/disease identification and early warning accuracy (Tao et al., 2025); and (iii) focusing on systematic integration and dynamic regulation mechanisms by embedding the detection model into intelligent greenhouse control systems to establish a complete decision-making closed loop from real-time identification and intelligent warning to coordinated regulation (Guo et al., 2020). With further validation across additional greenhouse structures and climatic regions, we will further validate the model on embedded edge platforms to assess real-world deployment performance. The proposed model may serve as a detection component in intelligent pest monitoring systems. Future work will focus on broader dataset expansion and embedded deployment evaluation.

## Data Availability

The raw data supporting the conclusions of this article will be made available by the authors, without undue reservation.
